# Outcomes by Mode of Transport of ST Elevation MI Patients in the United Arab Emirates

**DOI:** 10.5811/westjem.2017.1.32593

**Published:** 2017-03-13

**Authors:** Edward L. Callachan, Alawi A. Alsheikh-Ali, Satish Chandrasekhar Nair, Stevan Bruijns, Lee A. Wallis

**Affiliations:** * University of Cape Town, Department of Surgery, Division of Emergency Medicine, Bellville, South Africa; † Mohammed Bin Rashid University of Medicine and Health Sciences, College of Medicine, Dubai, United Arab Emirates; ‡ Sheikh Khalifa Medical City, Institute of Cardiac Sciences, Abu Dhabi, United Arab Emirates; § Tawam Hospital-Johns Hopkins Medicine Affiliate, Clinical Research, Al Ain, United Arab Emirates

## Abstract

**Introduction:**

The purpose of this multicenter study was to assess differences in demographics, medical history, treatment times, and follow-up status among patients with ST-elevation myocardial infarction (STEMI), who were transported to the hospital by emergency medical services (EMS) or by private vehicle, or were transferred from other medical facilities.

**Methods:**

This multicenter study involved the collection of both retrospective and prospective data from 455 patients admitted to four hospitals in Abu Dhabi. We collected electronic medical records from EMS and hospitals, and conducted interviews with patients in person or via telephone. Chi-square tests and Kruskal–Wallis tests were used to examine differences in variables by mode of transportation.

**Results:**

Results indicated significant differences in modes of transportation when considering symptom-onset-to-balloon time (p < 0.001), door-to-balloon time (p < 0.001), and health status at six-month and one-year follow-up (p < 0.001). Median times (interquartile range) for patients transported by EMS, private vehicle, or transferred from an outside facility were as follows: symptom-onset-to-balloon time in hours, 3.1 (1.8–4.3), 3.2 (2.1–5.3), and 4.5 (3.0–7.5), respectively; door-to-balloon time in minutes, 70 (48–78), 81 (64–105), and 62 (46–77), respectively. In all cases, EMS transportation was associated with a shorter time to treatment than other modes of transportation. However, the EMS group experienced greater rates of in-hospital events, including cardiac arrest and mortality, than the private transport group.

**Conclusion:**

Our results contribute data supporting EMS transportation for patients with acute coronary syndrome. Although a lack of follow-up data made it difficult to draw conclusions about long-term outcomes, our findings clearly indicate that EMS transportation can speed time to treatment, including time to balloon inflation, potentially reducing readmission and adverse events. We conclude that future efforts should focus on encouraging the use of EMS and improving transfer practices. Such efforts could improve outcomes for patients presenting with STEMI.

## INTRODUCTION

Acute coronary syndrome (ACS) is a leading cause of morbidity and mortality worldwide,^1^ with approximately half of these deaths occurring in the prehospital setting.^2^ For patients presenting with ST-elevation myocardial infarction (STEMI), percutaneous coronary intervention (PCI) is recommended.^3^ The short- and long-term mortality of STEMI patients can be reduced with PCI and coronary artery bypass grafting (CABG),^4^ with studies suggesting that primary PCI reduces mortality and major adverse cardiovascular events, when compared with thrombolytic therapy.^5^,^6^ The updated 2015 guidelines of the American College of Cardiology/American Heart Association (ACC/AHA) and the 2013 guidelines from the European Society of Cardiology recommend a door-to-balloon (D2B) time of less than 90 minutes.^6^,^7^ When this goal is met, PCI for STEMI reduces mortality and morbidity.^8^

Advanced prehospital management by emergency medical services (EMS) plays a crucial role in facilitating access to care and reducing mortality rates for STEMI patients.^7^–^11^ Studies have shown that transport by EMS is associated with quicker treatment, including shorter symptom-onset-to-arrival time and door-to-reperfusion time, when compared to private transport.^12^,^13^ Several studies have found that among patients who underwent emergency angiography, D2B times were shorter in EMS-transported patients.^13^–^15^

With EMS transport, treatment decisions can be made more quickly and effectively, as EMS can perform a prehospital electrocardiogram (ECG) and alert the hospital that the patient is en route, thereby minimizing door-to-reperfusion times.^16^–^20^ Prehospital ECG may also detect signs of transient ischemia and arrhythmias, which may no longer be present when the patient receives the first in-hospital ECG.^21^,^22^

The purpose of this multicenter study was to assess differences in patient demographics, medical history, symptoms, treatment times, and follow-up status among patients transported via EMS vs. those using private transport or those who were transferred from other medical facilities.

## METHODS

### Sample and Study Setting

This study was set in the Emirate of Abu Dhabi, United Arab Emirates (UAE), where both government and private hospitals provide cardiac catheterization services. Government hospitals are operated by the Abu Dhabi Health Services Company, while the EMS is operated by the Abu Dhabi Police Emergency and Public Safety Department and staffed by paramedics and EMT’s. For patients with suspected ACS, 12-lead ECG is performed and interpreted by paramedics. This interpretation involves paramedics activating the receiving hospital catheterization lab through a central activation number. Patients who are transferred by EMS from non-PCI centers receive advanced life-support care, including cardiac care (e.g. arrhythmia management), but the responsibility for catheterization lab activation lies within the inter-hospital transfer pathway, and not the EMS.

**Population Health Research Capsule**

What do we already know about this issue?

In the Middle East, EMS is underutilized and despite improvements in cardiac care, patient education about signs and symptoms of ACS, and the importance of EMS use is inadequate

What was the research question?

Does the mode of transport for care of patients with ACS affect clinical outcomes?

What was the major finding of the study?

EMS use was associated with shorter treatment times and potentially reduced adverse outcomes in the hospital events and readmission.

How does this improve population health?

This study highlights the benefits of using EMS in ACS care and could raise awareness and potentially increase EMS use in the Middle East.

### Procedures

This was a retrospective review of EMS and hospital data. Data obtained through chart review were supplemented with prospectively collected follow-up data. The study was conducted over a period of 18 months, with follow-up interviews at 30 days, six months, and one year after initial discharge. We recorded mode of transport (EMS, private, or transferred from other medical facility) and in-hospital events for each patient, using electronic medical records from both EMS and hospitals. Data included sex, age, past medical history (including history of 11 related conditions, such as hypertension, angina, diabetes mellitus types 1 and 2, and stroke), time of arrival, pain on arrival, door-to-ECG time, door-to-catheterization lab arrival time, D2B time, symptom-onset-to-balloon-inflation time (total ischemic time), hospital events (including eight related events such as bypass surgery, reinfarction, and mortality), 30-day follow-up status, six-month follow-up status, and one-year follow-up status.

### Statistical Analysis

Sample descriptive statistics are reported elsewhere.^23^ We calculated inferential statistics to determine whether significant differences existed between EMS-transported and privately transported patients with respect to the variables of interest. The Kruskal–Wallis rank test was used to estimate differences in continuous variables (door-to-first ECG, door-to-catheterization lab arrival, D2B time, and symptom-onset-to-balloon time) between modes of transportation. All other variables were categorical and were compared with chi-squared tests of independence. We performed all analyses using SPSS Version 22.0 (IBM Corporation, Armonk, NY, USA). All *p* values ≤ 0.05 were considered significant.

## RESULTS

### Demographics and History

We enrolled 455 consecutive patients with STEMI treated at four public hospitals in Abu Dhabi. A minority of patients (n = 53, 12%) arrived via EMS, and the remainder via private transport (n = 274, 60%) or were transferred from other facilities (n = 128, 28%). The majority of patients were male (94%), and half (52%) were active smokers. The average age was 51 ± 11 years, with 13% of patients younger than 40 years.

We observed no significant differences with respect to in-hospital events and discharge outcomes according to age (*p* = 0.121). No significant differences were noted in variables of health history according to mode of transportation, indicating that previous conditions did not affect the choice of transportation method.

Patients who arrived via private transportation were significantly more likely (*p* = 0.005) to arrive after hours (between 5 p.m. and 8 a.m. or on weekends). Other modes of transportation were approximately equal with respect to after-hours arrival.

For all modes of transportation, a high percentage (97%) of patients reported experiencing pain on arrival. We observed no significant differences in pain on arrival as a predictor of mode of transport among the groups (*p* = 0.16).

### Time to Treatment

Door-to-ECG time was available for all patients, with the median time being four minutes (interquartile range [IQR] two to seven minutes). At the time of this study, a 12-lead ECG was repeated for all patients in triage, prior to transport to the catheterization lab. The median door-to-ECG time was significantly higher for patients who used private transportation and EMS (both five minutes; IQR 2–8 and 2–6, respectively) than for transfer patients (four minutes) (*p* = 0.005). A door-to-ECG time of 10 minutes or less was achieved in 89% (n = 405) of patients. It is important to note that patients were also transferred from smaller clinic-type centers, requiring confirmation of STEMI, thereby justifying the rationale for adding door-to-ECG as a variable.

Door-to-catheterization lab arrival time data were available for 99% (n = 450) of patients. We found that privately transported patients had the longest door-to-catheterization lab arrival time (median = 74 minutes); this duration was significantly higher than that noted for EMS or transfer patients (*p* < 0.001). There was no significant difference observed for door-to-catheterization lab arrival times between EMS-transported and transfer patients (median = 45 minutes [28, 69] and 36 minutes [23, 55], respectively) (*p=* 0.462).

D2B time data were available for 96% (n = 438) of patients, with 76% (n = 334) of patients having a D2B time of 90 minutes or less. Privately transported patients had the longest D2B time (median = 81 minutes [64, 105]), which was statistically significant when compared to other modes of transportation (*p* < 0.001). We observed no significant difference between EMS and transfer patient D2B times (median = 70 minutes [48,89] and 62 minutes [46,77], respectively). Results related to treatment times are summarized in Table 1.

There were significant differences between modes of transport with respect to symptom-onset-to-balloon times (*p* < 0.001). Patients transferred from other medical facilities had the highest symptom-onset-to-balloon time (median = 4.5 hours [IQR 3.0–7.5]). Patients transported by EMS (median = 3.1 hours [IQR 1.8–4.3]) and privately (median = 3.2 hours [IQR 2.1–5.3]) had significantly shorter symptom-onset-to-balloon times (Table 2).

### In-Hospital Events and Follow-Up

Data for in-hospital events were available for 97% of patients. For the entire cohort, the rates of in-hospital events were as follows: cardiac arrest, 8.0% (n = 37); intra-aortic balloon pump, 5.6% (n = 26); CABG, 3.7% (n = 17); death, 3.2% (n = 15); in-stent thrombus, 1.1% (n = 5); stroke, 0.6% (n = 3); reinfarction, 0.2% (n = 1); bleeding, 0.2% (n = 1).

We observed a significant difference between the three modes of transportation with regards to the percentage of patients who required bypass surgery during their hospital stay (*p* = 0.017). Of the 17 patients who required bypass surgery, 11.3% arrived via EMS, 2.6% arrived by private transport, and 3.1% were transferred (Table 3).

We did not observe any significant differences with respect to transportation when considering any of the seven other in-hospital events studied. Patient follow-up status was categorized as follows: (1) no data or missing record; (2) home at follow-up; (3) readmitted to the catheterization lab since last follow-up; and (4) new disease event (includes reinfarction, stroke, and angina) or death since last follow-up. These statistics should not be confused with discharge-to-home status after the initial STEMI event; such discharge data are not available for the present study. There were no significant differences in health status at 30-day follow-up, with 75.4% of patients at home.

Differences in at-home status between the modes of transportation were significant (*p* < 0.001). Of the 390 patients available for follow-up at six months (85.7% of the original sample), 268 (58.9%) were at home and 21 (4.6%) had been readmitted since the 30-day follow-up. Of the patients originally transported via EMS, 18 (34.0% of EMS sample) were at home at the six-month follow-up, compared to 180 of those privately transported (65.7% of private transport sample) and 70 of those originally transferred (54.7%). These differences were significant (*p* < 0.001).

At the one-year follow-up, such observations remained consistent, but with fewer follow-up records available. Of the patients originally transported via EMS, 20.8% were at home (79.2% of records unavailable), compared with 52.2% of those privately transported (41.2% of records unavailable) and 34.3% of those originally transferred (62.5% of records unavailable). These differences were significant (*p* < 0.001).

At the one-year follow-up of patients originally transported privately, 13 had been readmitted between six months and one year after the initial treatment; one transfer patient and no EMS-transported patients exhibited similar readmission. This difference could, however, reflect the decreased availability of data for the EMS and transfer groups. Results from 30-day and one-year follow-ups are summarized in Table 4. All follow-up data are provided for completeness, despite a considerable loss to follow-up at one year.

## DISCUSSION

In this prospective, multicenter study of patients presenting with STEMI to a large network of public hospitals in Abu Dhabi, we observed that time-sensitive processes of care differed significantly according to the mode of transportation to the ED. Overall, total ischemic time (symptom-onset-to-balloon) was shortest among patients arriving by EMS, and longest among those transferred from other facilities.

While in-hospital processes (door-to-ECG, catheterization lab, and balloon times) were shortest among those transferred from outside facilities, these were offset by longer prehospital transfer times. Patients transported by EMS experienced a total ischemic time that was 1.4 hours (84 minutes) shorter than those transferred from elsewhere. Additionally, D2B and door-to-catheterization lab arrival times were 11 and 20 minutes shorter, respectively, among EMS-transported patients than privately transported patients; these differences were statistically significant.

These results are consistent with previous research showing that EMS transport is associated with shorter symptom-onset-to-hospital arrival and D2B times.^12^,^13^ Although there was also a statistically significant difference in door-to-ECG times when comparing transferred patients to non-transferred (EMS and private), this difference amounted to only one minute. These findings highlight the need to improve prehospital transport networks for patients with STEMI, in addition to efforts that aim to streamline the in-hospital processes of care.

This finding is especially interesting in the context of the 2015 updated guidelines from the ACC/AHA, which recommend transferring STEMI patients promptly to achieve a D2B time of less than 90 minutes from arrival at the initial facility.^6^ Although all groups in this study had median D2B times within this recommended range, the 84-minute difference when considering time-to-balloon inflation from symptom onset shows that the mode of transportation is an important variable for timely STEMI care. This is most important among patients who must be transferred from non-PCI capable facilities.

It is worth noting that the rates of in-hospital events, including cardiac arrest and mortality, were higher among the EMS group than among the privately transported group. This finding is, perhaps, partly accounted for by the relatively sicker population that EMS is likely to engage; younger, healthier patients with the ability to transport themselves privately may constitute a higher percentage of privately transported patients. Hence, the older and sicker EMS group may have been at a greater initial risk for poorer outcomes. Owing to the nature of the data collected, however, this hypothesis cannot be confirmed; future research is required to understand the difference in outcomes observed in this study. Similarly, the follow-up results provide little room for clear interpretation, owing to the large percentage of loss to follow-up.

It is important to promote the use of EMS, particularly for ACS, among the general public, especially given recent findings indicating an underuse of EMS among ACS patients in the Arabian Gulf region.^24^,^25^ Other findings made in this study with respect to specific trends of EMS use may be relevant in the promotion of EMS. Privately transported patients were more likely to arrive after hours (i.e., at night and on weekends). Existing research suggests that cardiac patients may be reluctant to bother EMS providers, and tend to wait to seek treatment until they are certain that their symptoms warrant medical attention. Such observations could explain our findings with respect to after-hours EMS use; indeed, reluctance to engage EMS providers is likely to be exacerbated outside of normal business hours.^26^

Another possible explanation of this finding is an increased tendency to visit EDs during times when primary care physicians are unavailable, suggesting a lack of access to after-hours care for non-emergent medical concerns.^27^ However, given that all patients in this study had STEMI and that there was no significant difference in the proportion of patients who reported pain on arrival, this possibility is unlikely for our sample. Therefore, improving public utilization of after-hours EMS could reduce time to reperfusion among ACS patients.

Additionally, facilities without interventional cardiology services urgently need to improve policies for the transfer of STEMI patients. Al Habib et al. recently emphasized the fact that, in the Arabian Gulf region, many of the vehicles used to transfer patients from primary care clinics to hospitals lack the equipment and personnel necessary to provide adequate prehospital ACS care.^24^

In the UAE, the setting of the present study, medical services are more developed than in many areas of the region, suggesting that these issues may also need to be addressed outside the urban area. Without organized systems to provide prehospital ACS care, the existence of PCI-capable facilities may not lead to associated improvements in ACS outcomes. In particular, in countries where EMS services are new or newly developing, public awareness and perception of EMS resources may lag behind actual service availability.

Researchers elsewhere noted that, when transferring patients to PCI centers for treatment, the time required to begin the transfer can significantly delay the overall time to treatment.^28^ Our findings clearly show that transfer practices to government-operated hospitals in Abu Dhabi should be improved to ensure adequate care for STEMI patients. Increased resource availability and training of professionals qualified for prehospital ACS treatment and diagnosis could lead to reduced treatment times and improved outcomes. Better transfer practices, including faster recognition and transfer policies, are urgently needed.

## LIMITATIONS

Our data are subject to limitations, which should be accounted for when interpreting the findings. Notably, many patients in the original sample were unavailable for follow-up, making it difficult to draw conclusions regarding the long-term impact of the differences observed. Additionally, when considering transfer patients, we did not record the source or reason for transfer. These factors could affect both transportation decisions and treatment times. Medical professionals should consider such factors when making transfer and transportation decisions. Further, EMS use for coronary symptoms should be encouraged among the general public to improve the quality of clinical outcomes for patients presenting with STEMI.

## CONCLUSION

We observed significantly lower time from symptom onset to hospital arrival and PCI balloon inflation among patients transported by EMS when compared with those transported privately or transferred from another facility. These findings support previous research showing that EMS care of ACS patients facilitates a more efficient delivery of care. Future efforts to promote the use of prehospital ECG are still needed.

## Figures and Tables

**Table 1:** Statistics from door-to-ECG, door-to-catheterization lab, and door-to-balloon time in a study examining how mode of transportation affected clinical outcomes in STEMI patients.

Variable	Mode	N^a^	Median (IQR^b^)	p
Door-to-ECG				
	EMS	53	5 (2, 6)	0.005
	Private	274	5 (2, 8)	
	Transfer	128	4(2, 6)	
Door-to-catheterization lab arrival				
	EMS	51	45 (28, 69)	< 0.001
	Private	274	74 (55, 96)	
	Transfer	125	36 (23, 55)	
D2B				
	EMS	49	70 (48, 89)	< 0.001
	Private	265	81 (64, 105)	
	Transfer	124	62 (46, 77)	

*D2B*, door-to-balloon; *ECG*, electrocardiogram; *EMS*, emergency medical services; *IQR*, interquartile range; *STEMI*, ST-elevation myocardial infarction.

a Data were not available for all patients.

b Interquartile range (first quartile, third quartile).

**Table 2:** Symptom-onset-to-balloon time according to mode of transportation to the emergency department.

Mode	N^a^	Median	IQR^b^	Min	Max	p
EMS	49	3.1	1.8, 4.3	1.1	24	< 0.001
Private	268	3.2	2.1, 5.3	0.9	16.3	
Transfer	128	4.5	3.0, 7.5	1.5	19.0	

*EMS*, emergency medical services; *IQR*, interquartile range.

a Data were not available for all patients.

b Interquartile range (first quartile, third quartile).

**Table 3:** Cross-tabulation for mode of transport and in-hospital events (n = 455).

Event	EMS^a^	Private	Transfer	Total	p
CABG	6 (11.3%)	7 (2.6%)	4 (3.1%)	17 (3.7%)	0.017
IABP	5 (9.4%)	16 (5.8%)	4 (3.1%)	26 (5.7%)	0.296
REINF	0 (0%)	1 (0.4%)	0 (0%)	1 (0.2%)	0.875
Bleed	0 (0 %)	1 (0.4%)	0 (0%)	1 (0.2%)	0.875
Stent throm	1 (1.9%)	3 (1.1%)	1 (0.8%)	5 (1.1%)	0.915
Stroke	1 (1.9%)	2 (0.7%)	0 (0%)	3 (0.7%)	0.536
Arrest	7 (13.2%)	23 (8.4%)	7 (5.5%)	37 (8.1%)	0.281
Death	4 (7.5%)	7 (2.6%)	4 (3.1%)	15 (3.3%)	0.282

*EMS*, emergency medical services; *CABG*, coronary artery bypass grafting; *IABP*, intra-aortic balloon pump; *REINF*, reinfarction; *Bleed*, any kind of bleed; *Stent Throm*, formation of an in-stent thrombus; *Arrest*, cardiac arrest.

a Column values indicate the number of individuals from each corresponding mode of transport to experience a given in-hospital event, with the percentage indicating the proportion these individuals represent within each mode of transport.

**Table 4:** Cross-tabulation for mode of transport and 30-day and one-year status (n = 455).

	30-day			1-year		
	
	EMS	Private	Transfer	EMS	Private	Transfer
Death	0 (0 %)	0 (0%)	1 (0.8%)	0 (0 %)	5 (1.8%)	3 (2.3%)
Readmission	1 (1.9%)	19 (6.9%)	15 (11.1%)	0(0%)	6 (2.2%)	0 (0%)
Reinfarction	0 (0 %)	12 (2.4%)	0 (0 %)	0(0%)	6 (2.2%)	0 (0%)
Lost to follow-up	16 (30.2%)	60 (21.9%)	8 (6.2%)	42 (79.2)	105 (38.3%)	74 (57.8%)

*EMS*, emergency medical services.

Statuses (e.g., stroke) not listed were not relevant to any patients at follow-up.

a Column values indicate the number of patients exhibiting the relevant status at a given follow-up duration. All percentages reflect original, not follow-up, sample sizes.
